# Chromatin remodeler BRD9 represses transcription of PPARα target genes, including CPT1A to suppress lipid metabolism

**DOI:** 10.1016/j.jlr.2025.100874

**Published:** 2025-08-06

**Authors:** Itsuki Yokoseki, Masataka Nakano, Kiamu Kurosawa, Yuichiro Higuchi, Shotaro Uehara, Nao Yoneda, Hiroshi Suemizu, Tatsuki Fukami, Miki Nakajima

**Affiliations:** 1 Drug Metabolism and Toxicology, Faculty of Pharmaceutical Sciences, Kanazawa University, Kanazawa, Japan; 2 WPI Nano Life Science Institute (WPI-NanoLSI) Kanazawa University, Kanazawa, Japan; 3 Liver Engineering Laboratory, Department of Applied Research for Laboratory Animals, Central Institute for Experimental Medicine and Life Science (CIEM), Kawasaki, Japan

**Keywords:** PPARs, lipolysis and fatty acid metabolism, dyslipidemias, lipid droplets, transcription, carnitine palmitoyltransferase 1A, chromatin remodeling, switch/sucrose non-fermentable complex, bromodomain-containing protein 9

## Abstract

Peroxisome proliferator-activated receptor α (PPARα) regulates the transcription of fatty acid oxidation-related genes, such as carnitine palmitoyltransferase 1A (CPT1A), to maintain lipid homeostasis. Recent studies have suggested the involvement of switch/sucrose non-fermentable (SWI/SNF) complexes in nuclear receptor-mediated transcription. SWI/SNF complexes are chromatin remodeling factors classified into three complexes: canonical brahma-related gene 1-/brahma-associated factor (cBAF), polybromo BAF (PBAF), and non-canonical BAF (ncBAF). Among these, the key regulator of PPARα-mediated transcription remains unclear. In this study, we sought to clarify the significance of each SWI/SNF complex in PPARα-mediated transcription. Glycerol sedimentation assay revealed that PPARα interacts with ncBAF. The expression of multiple PPARα target genes, including CPT1A, was increased in HepG2 cells or primary human hepatocytes by inhibition or knockdown of bromodomain-containing protein 9 (BRD9), a specific subunit of ncBAF. Co-immunoprecipitation and pull-down assays demonstrated that PPARα interacts with ncBAF via BRD9. Chromatin immunoprecipitation- and formaldehyde-assisted isolation of regulatory elements-qPCR analyses revealed that BI-9564, an inhibitor of BRD9, can enhance the binding of PPARα to the PPRE on *CPT1A* by relaxing the chromatin structure. Interestingly, lipid accumulation in free fatty acid-treated HepG2 cells was attenuated by BI-9564 treatment, and the administration of BI-9564 to mice decreased their plasma triglyceride levels. Collectively, this study demonstrated that BRD9 negatively regulates PPARα-mediated transactivation and that inhibition of BRD9 can attenuate lipid accumulation by enhancing hepatic lipid metabolism. Thus, BRD9 could be considered a novel pharmacological target for dyslipidemia.

Dyslipidemia is a risk factor for various disorders, such as cardiovascular disease, acute pancreatitis, and hyperuricemia (1, 2). Currently, statin treatment, which lowers low-density lipoprotein cholesterol levels, is the first-choice therapy for dyslipidemia. It has reduced the annual rate of major vascular events by 20% (3). Despite the benefits of statin treatment, 15% of the patients discontinue statin use because of myopathy (4). Fibrates are alternatively used in such patients; however, a meta-analysis of several clinical trials has revealed that fibrates do not affect all-cause mortality (5).

Peroxisome proliferator-activated receptor α (PPARα) is a target protein of fibrates. It is a nuclear receptor highly expressed in the liver, heart, and kidneys. PPARα upregulates the enzymes involved in fatty acid metabolism, including carnitine palmitoyl transferase 1A (CPT1A), a rate-limiting enzyme of mitochondrial fatty acid β-oxidation, acyl-CoA oxidase 1, a rate-limiting enzyme of peroxisomal fatty acid β-oxidation, and fatty acid binding protein 1 (FABP1); thus, PPARα activation leads to increased lipid metabolism and decreased serum triglyceride levels (6, 7). The promoters of these genes contain peroxisomal proliferator response element (PPRE), to which PPARα binds as a heterodimer with retinoid X receptor α (RXRα) (6). PPARα is activated by various synthetic and endogenous ligands, including fibrates, WY-14643, fatty acids, and leukotrienes (8, 9). Ligand binding enhances the stability of the PPARα-RXRα heterodimer, promotes its translocation to the nucleus, and results in complex formation with coactivators such as PPARγ coactivator 1α (PGC1α) or p300 (10, 11). In addition to these coactivators, brahma-related gene 1-/brahma-associated factor (BAF) 60a, one of the subunits of the switch/sucrose non-fermentable (SWI/SNF) chromatin remodeling complex, has been reported to directly interact with PPARα to positively regulate fatty acid oxidation-related gene expression (12).

Chromatin remodeling complexes are involved in gene transcription by changing the nucleosome structure and position using ATP hydrolysis energy (13). The SWI/SNF family, a chromatin remodeling complex family, includes three complexes: canonical BAF (cBAF), polybromo-associated BAF (PBAF), and non-canonical BAF (ncBAF) (14). These complexes have several common subunits, including brahma-related gene 1 (BRG1), BAF155, and BAF60a, along with unique subunits specific to each complex, such as AT-rich interactive domain-containing protein (ARID) 1A/1B, cBAF-specific subunits, bromodomain-containing protein (BRD) 7, a PBAF-specific subunit, and BRD9, an ncBAF-specific subunit (14). Differences in subunit assembly allow these complexes to have different localizations in the genome (15).

The BRG1 and BAF60a subunits, which are common to the three SWI/SNF complexes, are known to interact with nuclear receptors, such as the estrogen receptor, glucocorticoid receptor, and vitamin D receptor (VDR), to activate their transactivity (16). Recent evidence shows that BRD7 (in PBAF) and BRD9 (in ncBAF) interact with VDR to positively and negatively modulate VDR-mediated transcriptional activation of its target genes, such as *NFKBIA* (17), indicating the possibility that each SWI/SNF complex plays a different role in transcriptional regulation. Qu *et al.* (18) have reported that knockout of *Arid1a*, a cBAF-specific subunit, resulted in decreased expression of fatty acid oxidation-related genes, which are known to be regulated by Pparα, in the mouse liver, suggesting that the functional role of cBAF in lipid metabolism has been partially clarified. However, the involvement of the other SWI/SNF complexes in lipid metabolism remains unclear. In this study, we sought to clarify the significance of each SWI/SNF complex in the regulation of lipid metabolism in the context of PPARα transcriptional activation.

## Materials and methods

### Chemicals and reagents

BI-9564 was kindly provided by Boehringer Ingelheim (Ingelheim am Rhein) via its open innovation platform opnMe, available at https://opnme.com. WY-14643 was purchased from Tokyo Chemical Industry. Aprotinin, leupeptin, (*p*-amidinophenyl)methanesulfonyl fluoride hydrochloride (*p*-APMSF), proteinase K, PMSF, Dri-chem triglycerides, LabAssay triglyceride, and LabAssay NEFA were purchased from Wako Pure Chemical Industries. Lipofectamine RNAiMAX, Silencer Select siRNA (s26525, siBRD7; s35295, siBRD9; negative control #1, siControl), and Dynabeads Protein G were purchased from Thermo Fisher Scientific (Waltham, MA). pcDNA3.1 vector was purchased from Promega. RNAiso, random hexamers, and ribonuclease A were purchased from TaKaRa. ReverTra Ace was purchased from Toyobo. Luna Universal qPCR Master Mix and micrococcal nuclease were purchased from New England Biolabs. Eurofin Genomics commercially synthesized all primers. FAOBlue was purchased from Funakoshi. Rabbit polyclonal antibody for human CPT1A (15184-1-AP), and rabbit polyclonal antibody for human BRD7 (51009-2-AP) were purchased from Proteintech. Rabbit polyclonal antibodies for human PPARα [GTX101098 (for Western blotting) and ab24509 (for chromatin immunoprecipitation)] were purchased from GeneTex and Abcam, respectively. Rabbit polyclonal antibody for human BRD9 (A303-781A) was purchased from Bethyl Laboratories. Rabbit polyclonal antibody for human GAPDH (NB100-56875) was purchased from Novus Biologicals. Mouse monoclonal antibody for FLAG tag (F3165) was purchased from Sigma Aldrich. Mouse monoclonal antibody for His tag (D291-3S) was purchased from Medical and Biological Laboratories. Mouse monoclonal antibody for ac-lysine (sc-32268) and mouse normal IgG (sc-2025) were from Santa Cruz Biotechnology. Rabbit normal IgG (148-09551) was from Wako Pure Chemical Industries. IRDye 680 goat anti-rabbit and mouse antibodies were obtained from LI-COR Biosciences. All other reagents and solvents used were of highest grade and obtained from commercial sources.

### Cell cultures

Human hepatocellular carcinoma-derived HepG2 cells (Riken Gene Bank; passage number 20–40) were cultured in Dulbecco’s modified Eagle medium (DMEM; Nissui Pharmaceutical) containing 0.1 mM non-essential amino acids (Invitrogen) and 10% FBS (Invitrogen). For treatment with WY-14643, cells were cultured in advanced DMEM (Thermo Fisher Scientific) containing 0.1 mM non-essential amino acids. Human embryonic kidney-derived HEK293T cells (American Type Culture Collection; passage number 10–20) were cultured in DMEM containing 4.5 g/L d-glucose and 10% FBS. Primary human hepatocytes (HC5-2) were purchased from Sekisui Xenotech and were cultured in the OptiCULTURE hepatocyte culture medium (Sekisui Xenotech).

HepaSH cells, which are primary hepatocytes isolated from highly immunodeficient NOG mice transplanted with human hepatocytes (lot #1301-NCp, a 12-year-old female Caucasian; Lonza) were prepared at the Central Institute for Experimental Medicine and Life Science (19). The replacement rate of human hepatocytes is >95%. For initial seeding, HepaSH cells were plated in William’s E medium (Thermo Fisher Scientific) devoid of phenol red, supplemented with 10% FBS, 5 μg/ml bovine insulin (Sigma Aldrich), 100 IU/ml penicillin, 100 μg/ml streptomycin, and 2 mM glutamine. After three hours of incubation, the medium was replaced with maintenance medium composed of William’s E medium devoid of phenol red, 100 nM dexamethasone, and Cocktail B from the Primary Hepatocyte Maintenance Supplements (CM4000, Thermo Fisher Scientific). All cells were cultured at 37°C under an atmosphere of 5% CO_2_ and 95% air.

### Preparation of primary mouse hepatocytes

Male C57BL/6J mice were housed in the institutional animal facility according to the National Institutes of Health Guide for Animal Welfare of Japan, as approved by the Institutional Animal Care and Use Committee of Kanazawa University (AP-173857), under a controlled environment (temperature 25 ± 1°C and 12-h light/12-h dark cycle) with ad libitum food and water. After mice were anesthetized with isoflurane, the liver was perfused with HEPES-buffered saline (HBS, pH 7.4) containing 1 mM EDTA at 2.5 ml/min. The portal vein was cut to extravasate blood. After 3 min, the liver was perfused with HBS containing collagenase type IV (Sigma-Aldrich) at a flow rate of 5 ml/min. The liver was carefully dissected and placed in a 10 cm dish containing HBS. The liver was then disrupted with fine-tip forceps. The dispersed cells were filtered through a 70 μm cell strainer. Dead cells and non-parenchymal cells were removed by centrifugation at 50 × g for 4 min at 4°C.

### Transfection of expression plasmids or siRNA

The FLAG-PPARα plasmid was constructed by cloning human PPARα cDNA into the pcDNA3.1 vector and adding a 3×FLAG sequence at the *N*-terminus. The expression plasmids for human BRD7-His and BRD9-His were constructed as previously described (20). For co-immunoprecipitation, HEK293T cells (2.5 × 10^5^ cells) were seeded in a 6-well plate. After 24 h, the cells were transfected with 1 μg of FLAG-PPARα plasmid along with 3 μg of either BRD7-His or BRD9-His plasmid using Lipofectamine 3000. For the knockdown study, HepG2 cells (5 × 10^5^ cells) were seeded in a 6-well plate and transfected with 5 nM siRNA using Lipofectamine RNAiMAX.

### Preparation of total RNA, cell homogenates, and nuclear extracts

Total RNA was extracted from the cells and murine hepatic samples using RNAiso, according to the manufacturer's protocols. For cell homogenate preparation, cells were resuspended in TGE buffer [10 mM Tris-HCl, 1 mM EDTA, and 20% glycerol, pH 7.4], subjected to three freeze-thaw cycles, and homogenized with ten strokes in a Teflon-glass homogenizer. Nuclear extracts were prepared following the method reported previously (21).

### Glycerol gradient density sedimentation

A linear 10–30% glycerol gradients were prepared in 11 × 34 mm centrifuge tubes (Beckman Coulter) by layering 300 μl of 30%, 25%, 20%, 15%, and 10% HEMG solution [25 mM HEPES, 25 mM MgCl_2_, 0.1 mM EDTA, with corresponding glycerol, pH 7.5] from bottom to top. The nuclear extract from HepG2 cells (3 mg protein) in 150 μl of HEMG (0% glycerol) was overlaid in the prepared tube and centrifuged using a TLS-55 rotor (Beckman Coulter) at 55,000 rpm for 8 h at 4°C. Each 100 μl fraction was carefully collected from the upper layer and subjected to SDS-PAGE and Western blotting.

### SDS-PAGE and Western blot analysis

Cell homogenates, nuclear extracts, or immunoprecipitants were separated by 7.5% (for PPARα, BRD7, BRD9, and CPT1A) or 10% (for GAPDH) SDS-PAGE and transferred to an Immobilon-P transfer membrane (Millipore). Membranes were probed with primary antibodies followed by fluorescent dye-conjugated secondary antibodies. The band intensities were quantified using the Odyssey Infrared Imaging System (LI-COR Biosciences). Protein levels of CPT1A were normalized to GAPDH. Western blot experiments were conducted with three independent replicates.

### Real-time RT-PCR

cDNA was synthesized from total RNA using ReverTra Ace and random hexamers according to the manufacturer’s instructions. PCR reactions consisted of 1 μl of reverse-transcribed product, 10 pmol of each primer, and 10 μl of Luna Universal qPCR Master Mix in a final volume of 20 μl. The gene expression levels were quantified using a standard curve generated from serially diluted samples and normalized to the level of GAPDH. All real-time RT-PCR experiments were performed with at least three independent replicates. Real-time RT-PCR was performed on a QuantStudio 1 system (Thermo Fisher Scientific). Primer sequences and PCR conditions are provided in Table 1.

**Table 1:** Primers sequences and conditions for real-time PCR

Primer	Sequence (5′ to 3′)	Annealing Temperature (°C)	Extension Time (s)	Step
hCPT1A S (48)	GCC TCG TAT GTG AGG CAA AA	64	20	2
hCPT1A AS (48)	TCA TCA AGA AAT GTC GCA CG			
hPDK4 S (26)	TGG AGC ATT TCT CGC GCT AC	58	20	2
hPDK4 AS (26)	ACA GGC AAT TCT TGT CGC AAA			
hPLIN2 S (26)	TGA GAT GGC AGA GAA CGG TGT G	58	20	2
hPLIN2 AS (26)	GGC ATT GGC AAC AAT CTG AGT			
hCPT2 S	CGG AGT CTC GAG CAG ATA GG	55	20	2
hCPT2 AS	GGA AAA GAA CTG CAT GAG CA			
hSLC25A20 S	GGG GTC ACT CCC ATG TTT G	55	20	2
hSLC25A20 AS	TGT GGT GAA TAC GCC AGA TAA C			
hFABP1 S	TCA AGG GGG TGT CGG AAA TC	55	20	2
hFABP1 AS	GTG ATT ATG TCG CCG TTG AGT TC			
hGAPDH S (50)	CCA GGG CTG CTT TTA ACT C	64	20	2
hGAPDH AS (50)	GCT CCC CCC TGC AAA TGA			
mCpt1a S	GCA TAC CAA AGT GGA CCC CT	64	20	2
mCpt1a AS	TGC TCT GCA AAC ATC CAG CC			
mCpt2 S	ACC AAG GCT AGA CAC CTC CT	58	20	2
mCpt2 AS	GCC CAG ACA TCT CGG TTC TC			
mSlc25a20 S	GGA GTC ACC CCT ATG TTC GC	58	20	2
mSlc25a20 AS	ATC CGT TCT CCA GGG GTC AT			
mFabp1 S	GGA AGG ACA TCA AGG GGG TG	58	20	2
mFabp1 AS	TCA CCT TCC AGC TTG ACG AC			
mGapdh S (51)	TCA CCA GGG CTG CCA TTT G	64	20	2
mGapdh AS (51)	CTC ACC CCA TTT GAT GTT AGT			
CPT1A +2432 S (28)	AAC TCG CTC CGG AAG GTC TC	57	30	2
CPT1A +2634 AS (28)	GCT TGG AAA GCG TCC TCG GAA GG			

Dushay *et al.*, 2010 (48), Rakhshandehroo *et al.*, 2009 (26), Tachibana *et al.*, 2005 (49), Tsuchiya *et al.*, 2004 (50), Akai *et al.*, 2007 (51), Napal et al., 2005 (28).

S: sense primer. AS: anti-sense primer.

### Co-immunoprecipitation assay

HEK293T cells overexpressing FLAG-PPARα and BRD7/9-His were harvested and lysed in ice-cold lysis buffer [20 mM HEPES, 0.3 M NaCl, 0.2 mM EDTA, 0.5% NP-40, 15% glycerol, protease inhibitor cocktail (PIC) (2 μg/ml aprotinin, 2 μg/ml leupeptin, and 100 μM *p*-APMSF in water), pH 8.0]. Anti-His tag antibody was bound to Dynabeads Protein G. Cell lysates were then added to the antibody-bead complex and incubated for 2 h at room temperature. After washing three times with IP buffer [PBS containing 2 mM EDTA and 1% Triton X-100], the immunoprecipitants were eluted with SDS-PAGE sample buffer for Western blot analysis.

### Pull-down assay

Anti-FLAG tag antibody was bound to Dynabeads Protein G. HEK293T cell lysates overexpressing FLAG-PPARα were added to the antibody-bead complex and incubated for 2 h at room temperature. After washing three times with IP buffer, the protein-beads complex was incubated with His tag-fused BRD7 or BRD9 proteins purified from HEK293T cell lysates overexpressing BRD7-His or BRD9-His, respectively, for 12 h at 4°C. The immunoprecipitants were washed three times with IP buffer, and bound proteins were eluted with SDS-PAGE sample buffer for Western blot analysis.

### Chromatin immunoprecipitation (ChIP)

HepG2 cells were fixed with 1% paraformaldehyde in PBS for 30 min at room temperature. After the cells were collected, they were frozen, thawed, and resuspended in ChIP lysis buffer [5 mM PIPES, 200 mM KCl, 1.5 mM MgCl_2_, 1 mM CaCl_2_, 5% sucrose, 0.5% NP-40, PIC] for 15 min on ice. The sample was sonicated (3 cycles, 7 s each), and chromatin was digested with micrococcal nuclease and RNase A for 20 min at 37°C. Following centrifugation at 8,500 rpm for 8 h at 4°C, the supernatant (containing 8 μg DNA) was incubated with 10 μl Dynabeads Protein G and 2 μg anti-PPARα antibody for 2 h at room temperature. The bead-chromatin-antibody complexes were washed four times with wash buffer [10 mM Tris-HCl, 140 mM NaCl, 1 mM EDTA, 0.5% Triton X-100, 1% glycerol, and 0.01% SDS, pH 8.0] and twice with LiCl buffer [10 mM Tris-HCl, 250 mM LiCl, 1 mM EDTA, and 0.5% Triton X-100, pH 8.0]. Immunoprecipitants were resuspended in elution buffer [10 mM Tris-HCl, 300 mM NaCl, 5 mM EDTA, 1% SDS, and 0.8 mg/ml proteinase K, pH 8.0] and incubated for 1 h at 55°C, followed by 4 h at 65°C. DNA was purified by phenol-chloroform extraction and analyzed by real-time PCR with primers and conditions listed in Table 1.

### Formaldehyde-assisted isolation of regulatory elements

Formaldehyde-assisted isolation of regulatory elements was performed based on the method described by Giresi *et al.* (22), with minor adjustments. In brief, HepG2 cells (1 × 10^6^) were exposed to 1% paraformaldehyde in PBS for 2 min at room temperature. After collection, cells were lysed in 10 volumes of cold buffer [10 mM Tris-HCl, 0.1 M NaCl, 1 mM EDTA, 2% Triton X-100, 1% SDS, 1 mM PMSF, pH 8.0] and subjected to sonication (10 cycles, 30 s each). The lysate was centrifuged at 15,000 rpm for 5 min at 4°C. DNA was extracted from the supernatant using phenol-chloroform and analyzed by real-time PCR with primers and conditions listed in Table 1.

### Evaluation of fatty acid β-oxidation activity

HepG2 cells were pre-treated with 20 μM BI-9564 for 12 h, followed by co-treatment with 10 μM WY-14643 and 20 μM BI-9564 for an additional 48 h. After these treatments, the cells were then incubated with 0.5 μM FAOBlue, a fatty acid oxidation detection reagent. After incubation for 30 min, fluorescence was measured at 405 nm excitation and 450 nm emission using a Spark 10M multimode microplate reader (TECAN).

### Oil red O staining

The cells were pre-treated with 20 μM BI-9564 for 12 h, followed by co-treatment with 10 μM WY-14643 and 20 μM BI-9564 for 36 h. Subsequently, the cells were co-treated with 10 μM WY-14643, 20 μM BI-9564, and 400 μM FFAs (2:1 ratio of oleic acid to palmitic acid) for 12 h. The cells were then washed twice with PBS and fixed with 10% formaldehyde for 1 h at room temperature. Intracellular lipid droplets were stained with oil red O solution (oil red O-saturated 60% isopropanol solution) for 10 min. After washing twice with 60% isopropanol, the cells were observed under a microscope (TE2000-U, NICON) and photographed. To quantify the intracellular lipid droplet levels, cellular Oil Red O was eluted with isopropanol, and the absorbance was measured at 492 nm.

### Measurement of intracellular triglyceride level

The cells were treated as described in Section 2.14. After treatment, the cells were washed twice with PBS and lysed in cold lysis buffer [50 mM Tris-HCl (pH 7.4), 150 mM NaCl, 1% NP-40, 0.1% SDS, 1 mM EDTA, and 10 mM NaF]. Intracellular triglyceride levels were measured using the LabAssay triglyceride, following the manufacturer's instructions. The triglyceride levels were normalized to the protein concentration of the cell lysates.

### Administration of WY-14643 and/or BI-9564 to C57BL/6J mice and measurement of plasma concentrations of triglyceride and non-esterified fatty acid

Male C57BL/6J mice (8 weeks old, 25.4 ± 1.4 g) were housed in a temperature- and humidity-controlled environment with a 12-h light/dark cycle and had ad libitum access to standard chow (PicoLab Rodent Diet 20, Lab Diet) and water. Mice were intraperitoneally administered 10 mg/kg WY-14643 and/or 100 mg/kg BI-9564, both dissolved in 1% carboxymethyl cellulose for 4 days. Control mice received 1% CMC alone under the same conditions. After fasting for 6 h following the final dose, mice were euthanized for liver and blood collection. Plasma triglycerides and NEFA were measured using Dri-chem Triglycerides and LabAssay NEFA, respectively.

### Data analyses

Data distribution was evaluated using the Shapiro–Wilk test, and variance homogeneity was examined with Levene’s test. For datasets that met the assumptions of normality and homoscedasticity, one-way analysis of variance (ANOVA) followed by Tukey’s test was applied. For non-normally distributed data, the Kruskal–Wallis test followed by Dunn’s test was applied. In cases of unequal variances, the Games-Howell test was employed. A *P*-value < 0.05 was considered statistically significant.

## Results

### BRD9 inhibition enhances PPARα-mediated induction of lipid metabolism genes

**Fig. 1:**
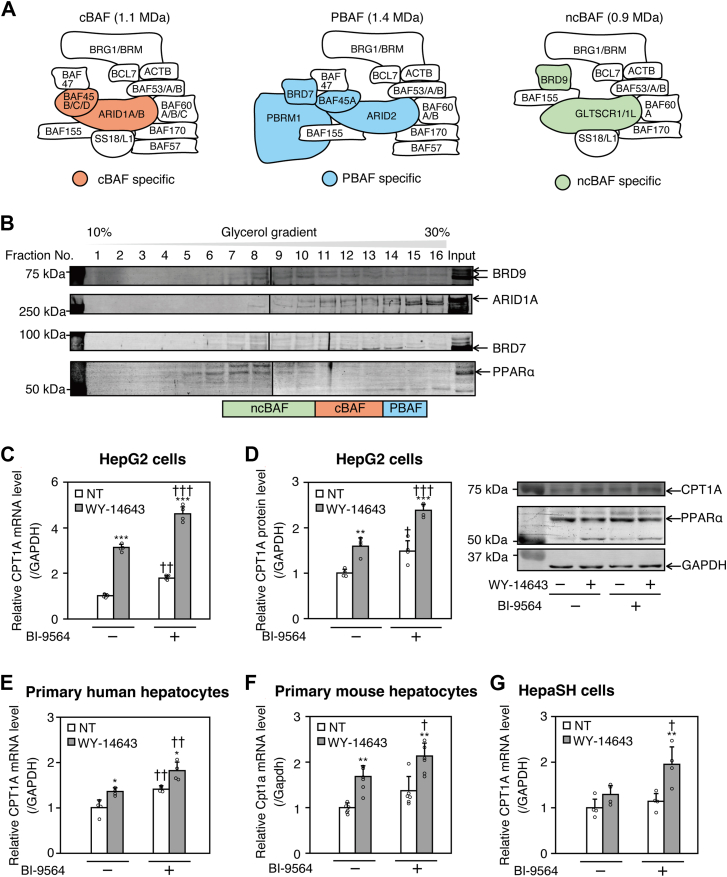
Inhibition of BRD9 enhances the induction of PPARα-downstream gene CPT1A by WY-14643. A: Schematic image of mammalian cBAF, PBAF, and ncBAF (47). B: Glycerol gradient density sedimentation of the nuclear extract of HepG2 cells. Subunits of each SWI/SNF complex and PPARα protein were detected by Western blot analysis (C–G) HepG2, PHH, PMH, or HepaSH cells were pre-treated with 20 μM BI-9564. After 12 h, the cells were co-treated with 10 μM WY-14643 and 20 μM BI-9564 for 48 h. Human (C and E) and mouse (F) CPT1A mRNA levels were determined using real-time RT-PCR. (D) CPT1A, PPARα, and GAPDH protein levels in HepG2 cells were determined by Western blotting. Each column represents the mean ± SD (n = 4). Western blot experiments were conducted with three independent replicates. In panel (C), data were analyzed using one-way ANOVA followed by the Games-Howell test. In panel (F), data were analyzed using the Kruskal–Wallis test followed by Dunn’s test. Data in all other panels were analyzed by one-way ANOVA followed by Tukey’s test. ∗*P* < 0.05, ∗∗*P* < 0.01, and ∗∗∗*P* < 0.001, compared with NT. ^†^*P* < 0.05, ^††^*P* < 0.01 and ^†††^*P* < 0.001, compared with BI-9564 (−). NT: non-treatment.

The SWI/SNF complexes cBAF, PBAF, and ncBAF consist of common or unique components (Fig. 1A) and have different molecular weights (1.1, 1.4, and 0.9 MDa, respectively). Glycerol gradient density sedimentation (14) can be used to separate these complexes. To determine which SWI/SNF complex interacts with PPARα, we performed a glycerol gradient centrifugation using a nuclear extract of HepG2 cells. As shown in Fig. 1B, BRD9 in ncBAF, ARID1A in cBAF, or BRD7 in PBAF were included in fractions 6–10, 12–14, or 14–16, respectively, and PPARα was detected in the fractions 5–10, which overlapped with the fractions including ncBAF, suggesting that PPARα is co-localized with ncBAF. The specificity of the PPARα band detected by the anti-PPARα antibody (GTX101098) was confirmed by PPARα-targeting knockdown by siRNA and validated with an alternative anti-PPARα antibody (ab24509), as shown in supplemental Fig. 1.

Increased expression of *CPT1A*, a major PPARα-downstream gene, has been reported to suppress hepatocyte lipid accumulation and plasma triglycerides (23). We therefore investigated the effect of ncBAF on the induction of CPT1A expression by WY-14643, a PPARα agonist. HepG2 cells were co-treated with WY-14643 and BI-9564, a specific BRD9 inhibitor. The mRNA (Fig. 1C) and protein (Fig. 1D) levels of CPT1A were significantly increased by a single treatment of WY-14643 or BI-9564. Co-treatment with WY-14643 and BI-9564 resulted in a further increase in CPT1A mRNA and protein levels. The PPARα protein level was not altered by BI-9564 and/or WY-14643 (Fig. 1D). These results suggest that BRD9 negatively regulates PPARα-mediated transcriptional activation of *CPT1A*.

In addition, CPT1A expression levels were evaluated in primary human hepatocytes (PHH) and primary mouse hepatocytes (PMH) to confirm whether the BRD9 inhibitor enhances CPT1A induction in normal hepatocytes. Consistent with the results in HepG2 cells, the induction of CPT1A mRNA by WY-14643 was enhanced by treatment with BI-9564 (Fig. 1E, F). Because the responsiveness of PHH to PPARα ligands can vary depending on the lot, we used HepaSH cells, hepatocytes derived from humanized liver mice that minimize lot-to-lot variability (19), to confirm the reproducibility of the findings. Consistent with the observations in Figure 1C, E and F, BI-9564 further enhanced the induction of CPT1A expression in HepaSH cells, although the overall induction was weaker than PHH (Fig. 1G). These results suggest that BRD9 negatively regulates PPARα-mediated transcriptional activation of *CPT1A*.

**Fig. 2:**
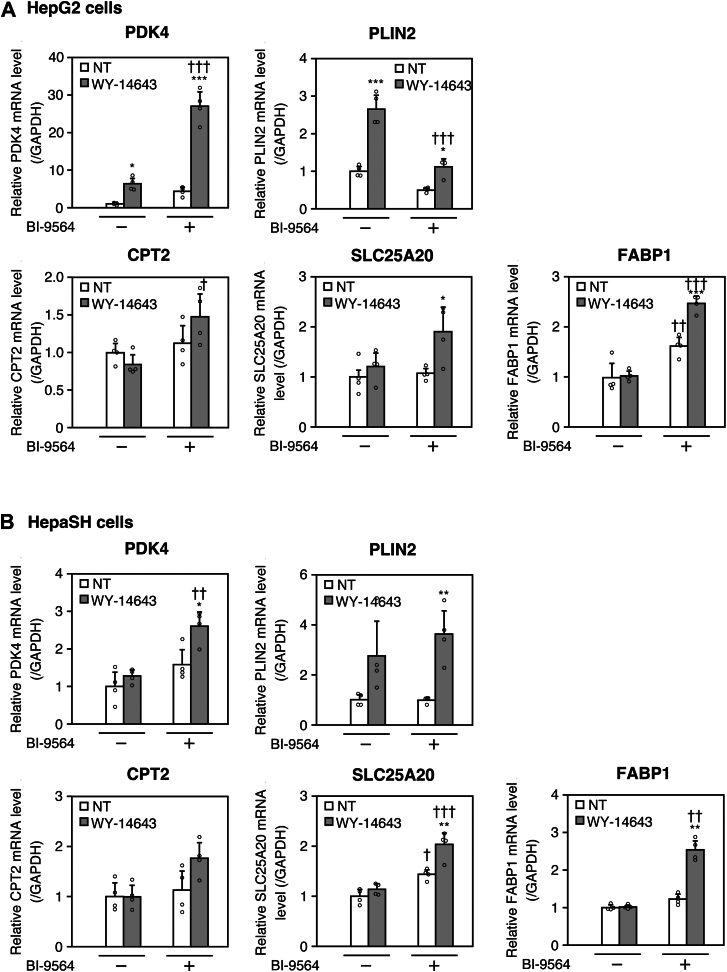
Effects of WY-14643 and BI-9564 on the mRNA levels of additional PPARα target genes in HepG2 and HepaSH cells. HepG2 (A) and HepaSH (B) cells were pre-treated with 20 μM BI-9564. After 12 h, the cells were co-treated with 10 μM WY-14643 and 20 μM BI-9564 for 48 h. PDK4, PLIN2, CPT2, SLC25A20, and FABP1 mRNA levels were determined using real-time RT-PCR. Each column represents the mean ± SD (n = 4). Data in all other panels were analyzed by one-way ANOVA followed by Tukey’s test. ∗*P* < 0.05, ∗∗*P* < 0.01, and ∗∗∗*P* < 0.001, compared with NT. ^†^*P* < 0.05, ^††^*P* < 0.01, and ^†††^*P* < 0.001, compared with BI-9564 (−). NT: non-treatment.

We next examined the effects of BI-9564 on the expression of additional PPARα downstream genes, pyruvate dehydrogenase kinase 4 (PDK4), perilipin 2 (PLIN2), CPT2, solute carrier family 22 member 20 (SLC25A20), and FABP1 (24). In HepG2 cells, WY-14643 significantly increased the expression of PDK4 and PLIN2, and co-treatment with BI-9564 further enhanced PDK4 induction but attenuated PLIN2 induction (Fig. 2A). Although treatment with WY-14643 alone did not increase the expression of CPT2, SLC25A20, and FABP1, this was likely because the concentration of WY-14643 (10 μM) is lower than that typically reported in other studies (25, 26). Nevertheless, co-treatment with BI-9564 led to increased expression of these genes (Fig. 2A). In HepaSH cells, while treatment with either WY-14643 or BI-9564 alone caused little or no induction of these PPARα target genes, co-treatment with WY-14643 and BI-9564 significantly increased their expression (Fig. 2B). These findings suggest that BRD9 inhibition generally enhances the induction of PPARα downstream genes, although some exceptions may arise due to differences in underlying regulatory mechanisms.

### Knockdown of BRD7 or BRD9 enhances CPT1A induction by WY-14643 in HepG2 cells

**Fig. 3:**
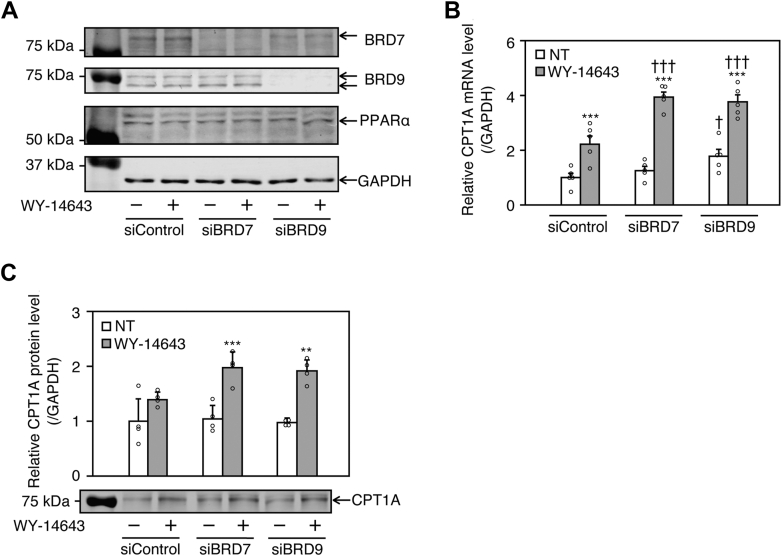
Effects of BRD7 and BRD9 knockdown on CPT1A expression in WY-14643-treated HepG2 cells. A–C: HepG2 cells were transfected with siRNA against BRD7 (siBRD7) or BRD9 (siBRD9). After 24 h, the cells were treated with 30 μM WY-14643 for 48 h. A: BRD7, BRD9, PPARα, and GAPDH protein levels were determined using Western blot analysis. B and C: CPT1A mRNA and protein levels were determined using real-time RT-PCR and Western blotting analysis, respectively. Each column represents the mean ± SD (n = 4). Western blot experiments were conducted with three independent replicates. Data in all panels were analyzed by one-way ANOVA followed by Tukey’s test. ∗∗*P* < 0.01 and ∗∗∗*P* < 0.001, compared with NT, ^†^*P* < 0.05 and ^†††^*P* < 0.001, compared with siControl. NT: non-treatment.

Next, the expression level of CPT1A was evaluated in siRNA for BRD7 (siBRD7)- or siBRD9-transfected and WY-14643-treated HepG2 cells. Upon transfection with siBRD7 or siBRD9, the BRD7 or BRD9 protein level was markedly decreased both with and without WY-14643 (Fig. 3A). PPARα protein levels remained unchanged by BRD7 or BRD9 knockdown (Fig. 3A). WY-14643 treatment significantly upregulated CPT1A mRNA expression. This upregulation was further enhanced by BRD7 or BRD9 knockdown (Fig. 3B). In addition, basal CPT1A mRNA levels were significantly increased following BRD9 knockdown (Fig. 3B). At the protein level, WY-14643 increased CPT1A expression in siControl-transfected cells, although this increase was not statistically significant. However, in cells transfected with siBRD7 or siBRD9, WY-14643 induced a significant increase in CPT1A protein levels (Fig. 3C). These results suggest that PBAF as well as ncBAF, negatively regulates PPARα-mediated transcriptional activation of CPT1A.

### BRD9 directly interacts with PPARα

**Fig. 4:**
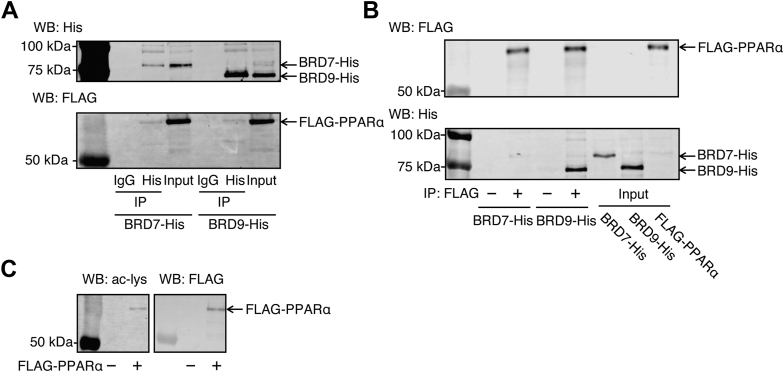
BRD9 directly interacts with PPARα. A: Co-immunoprecipitation assay to examine the interaction between PPARα and BRD9 was performed. HEK293T cells were transfected with FLAG-PPARα plasmid together with the BRD7-His or BRD9-His plasmids using Lipofectamine 3000. Cell lysates were subjected to co-immunoprecipitation with His-tag antibody. B: Direct interaction between PPARα and BRD9. HEK293T cells were individually transfected with FLAG-PPARα, BRD7-His, or BRD9-His plasmids. Affinity purification was conducted using cell lysates, and the purified FLAG-PPAR protein was incubated with purified BRD7/9-His proteins. C: Western blotting to analyze lysine acetylation of PPARα. Lysate of FLAG-PPARα plasmid-transfected HEK293T cells was subjected to Western blotting using anti-acetylated lysine and anti-FLAG antibodies. A smaller amount of protein (10 μg) was loaded compared to Figure 4A (30 μg). Western blot experiments were conducted with three independent replicates.

BRD9, an ncBAF-specific subunit, has been reported to interact with VDR and pregnane X receptor (PXR), members of the nuclear receptor superfamily to which PPARα also belongs (17, 19). To examine whether BRD9 interacts with PPARα, a co-immunoprecipitation assay was performed using lysates from FLAG-PPARα and BRD9-His-expressing HEK293T cells. FLAG-PPARα was detected in BRD9-His immunoprecipitant (Fig. 4A), suggesting that PPARα interacts with ncBAF. Unexpectedly, FLAG-PPARα was also detected in the BRD7-His immunoprecipitant, suggesting that PPARα also interacts with PBAF. To further confirm the direct interaction between PPARα and BRD9, a pull-down assay was performed using an anti-FLAG antibody. The results showed stronger band intensity for BRD9-His compared to BRD7-His, while FLAG-PPARα levels remained consistent (Fig. 4B). These results demonstrated that PPARα interacts directly with ncBAF through BRD9, and that their interaction is stronger than that between PPARα and BRD7. BRD7 and BRD9 contain bromodomains that recognize and bind acetylated lysine residues in proteins (27). To examine whether PPARα has acetylated lysine residue(s), Western blot analyses were performed using anti-acetylated lysine and anti-FLAG antibodies. A band corresponding to FLAG-PPARα was detected with both antibodies (Fig. 4C), suggesting that the FLAG-PPARα protein has acetylated lysine residue(s). These results indicate that the bromodomain of BRD7 or BRD9 interacts with acetylated PPARα protein.

### BRD9 inhibition enhances the binding of PPARα to the intronic PPRE of the *CPT1A* gene in HepG2 cells

**Fig. 5:**
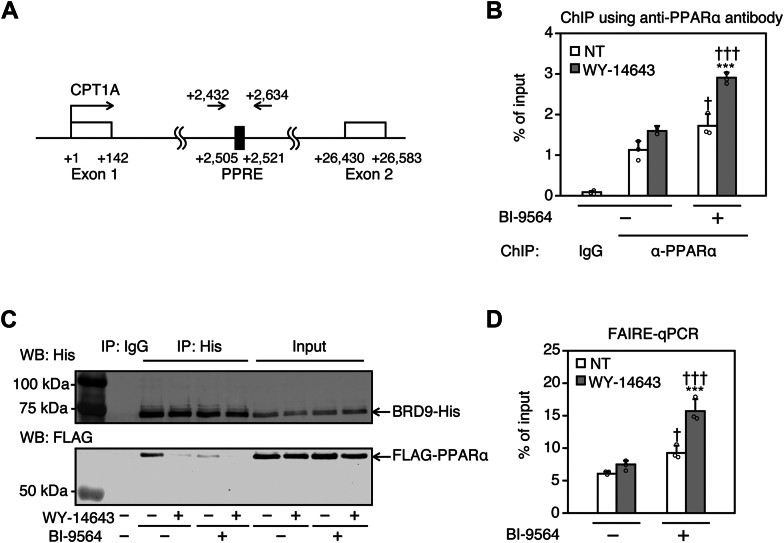
Effects of WY-14643 and/or BI-9564 treatment on the binding of PPARα and BRD9 and on the chromatin accessibility around the *CPT1A* intronic peroxisomal proliferator response element (PPRE). A: Scheme of the *CPT1A* gene. B: Binding of PPARα to *CPT1A* intronic PPRE. HepG2 cells were pre-treated with 20 μM BI-9564. After 12 h, the cells were co-treated with 10 μM WY-14643 and 20 μM BI-9564 for 48 h. A ChIP assay using an anti-PPARα antibody was performed, and the purified DNA was analyzed by real-time PCR targeting *CPT1A* intronic PPRE. C: The interaction between PPARα and BRD9. HEK293T cells were transfected with FLAG-PPARα and BRD9-His plasmids using Lipofectamine 3000, and treated with 30 μM WY-14643 and/or 40 μM BI-9564 for 48 h. The cell lysates were co-immunoprecipitated with anti-His-tag antibody. FLAG-PPARα and BRD9-His were detected by Western blotting. D: Chromatin accessibility around *CPT1A* intronic PPRE. HepG2 cells were pre-treated with 20 μM BI-9564. After 12 h, the cells were co-treated with 10 μM WY-14643 and 20 μM BI-9564 for 48 h; a FAIRE assay was then performed on the cells. Purified DNA was analyzed by real-time PCR targeting the *CPT1A* intronic PPRE. Each column represents the mean ± SD (n = 3). Data in all panels were analyzed by one-way ANOVA followed by Tukey’s test. ∗∗∗*P* < 0.001, compared with NT; ^†^*P* < 0.05 and ^†††^*P* < 0.001, compared with BI-9564 (−). NT: non-treatment.

Next, we investigated the mechanism by which PPARα-mediated transcription of *CPT1A* was up-regulated by BI-9564. There is a PPRE in intron 1 of human *CPT1A* (Fig. 5A) (28). A ChIP assay was performed to investigate whether BRD9 affects the binding of PPARα to this region. Compared with IgG, enrichment of intronic PPRE in *CPT1A* was observed in the immunoprecipitant of the PPARα antibody. This enrichment was enhanced by WY-14643, though the difference was not statistically significant (Fig. 5B). BI-9564 treatment significantly increased the enrichment, regardless of WY-14643 treatment. This result suggests that treatment with BI-9564 increases PPARα binding to *CPT1A* intronic PPRE. A co-immunoprecipitation study indicated that the interaction between BRD9 and PPARα was markedly attenuated by treatment with WY-14643 or BI-9564 (Fig. 5C), suggesting that CPT1A expression increased by the co-treatment of WY-14643 and BI-9564 was caused by the dissociation of PPARα from ncBAF and the subsequent binding of PPARα to PPRE. To examine the possibility that ncBAF inhibits PPARα binding to *CPT1A* intronic PPRE by modulating the chromatin structure, a FAIRE-qPCR was performed. The results showed that chromatin accessibility around the *CPT1A* intronic PPRE was not increased by treatment with WY-14643, but was increased by BI-9564, both with and without BI-9564 (Fig. 5D). These results suggest that ncBAF ameliorates the binding of PPARα to *CPT1A* intronic PPRE by condensing the chromatin structure. Further, the BRD9 inhibitor can enhance the ligand-induced binding of PPARα to *CPT1A* intronic PPRE.

### BI-9564 decreases lipid accumulation in HepG2 cells and plasma triglyceride levels in mice

**Fig. 6:**
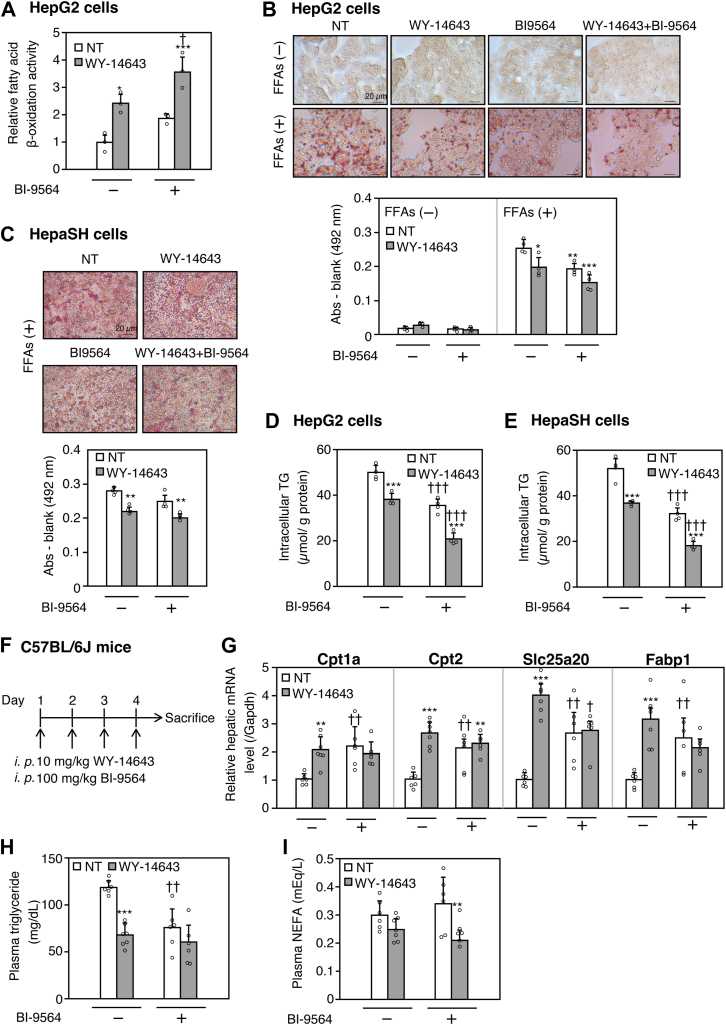
Effects of WY-14643 and/or BI-9564 treatment/administration on lipid accumulation in HepG2 and HepaSH cells and hepatic Cpt1a expression, plasma triglyceride, or plasma non-esterified fatty acid (NEFA) levels in C57BL/6J mice. A: Fatty acid β-oxidation activity in HepG2 cells treated with WY-14643 and/or BI-9564. The cells were treated with 0.5 μM FAOBlue for 30 min, and fluorescence at 405 nm excitation and 450 nm emission were measured. B, C: Lipid accumulation in HepG2 and HepaSH cells treated with WY-14643 and/or BI-9564 in the presence of 400 μM free fatty acids (FFAs) was evaluated by oil red O staining. Intracellular lipid droplets were evaluated by measuring absorbance at 492 nm; representative photomicrographs are shown. Each column represents the mean ± SD (n = 4). D, E: Intracellular triglyceride levels in HepG2 and HepaSH cells treated with WY-14643 and/or BI-9564 in the presence of 400 μM FFAs were evaluated by LabAssay triglyceride kit. Each column represents the mean ± SD (n = 4). F–I: C57BL/6J mice (8 weeks, male) were administered with 10 mg/kg WY-14643 and/or 100 mg/kg BI-9564 once a day for 4 days. Six hr after the last administration, the liver and plasma were collected. Cpt1a mRNA (G), plasma triglyceride (H), and plasma NEFA (I) levels were determined using real-time RT-PCR, Dri-chem triglycerides, and LabAssay NEFA, respectively. Each column represents the mean ± SD (n = 6 for all groups except WY-14643, n = 7). In panel (C), data were analyzed using one-way ANOVA followed by the Games-Howell test. Data in all other panels were analyzed by one-way ANOVA followed by Tukey’s test. ∗*P* < 0.05, ∗∗*P* < 0.01, and ∗∗∗*P* < 0.001, compared with NT, ^†^*P* < 0.05 and ^††^*P* < 0.01, compared with BI-9564 (−). NT: non-treatment.

We investigated the effects of BI-9564-induced activation of PPARα target genes, including CPT1A, on lipid metabolism in hepatocytes. CPT1A is known to be a rate-limiting enzyme in mitochondrial fatty acid β-oxidation. Consistent with the observed increase in CPT1A expression, HepG2 cells demonstrated a significant rise in mitochondrial fatty acid β-oxidation activity when treated with WY-14643. This effect was further enhanced by co-treatment with BI-9564 (Fig. 6A). To assess the influence of WY-14643 and BI-9564 on intracellular lipid accumulation, HepG2 cells were exposed to free fatty acids (FFAs) and subsequently stained with oil Red O. Intracellular lipid droplets were detected in FFA-treated cells, whereas lipid droplet accumulation was significantly decreased by BI-9564 or WY-14643 treatment; this decrease was enhanced by the combined treatment of BI-9564 and WY-14643 (Fig. 6B). Next, to further validate these findings in a more physiologically relevant model, we performed oil red O staining in WY-14643- and/or BI-9564-treated HepaSH cells. Treatment with BI-9564 alone did not lead to a significant reduction in lipid accumulation by FFAs (Fig. 6C). However, co-treatment with WY-14643 and BI-9564 resulted in the most pronounced reduction in intracellular lipid droplets, similar to the effect observed in HepG2 cells. Consistent with these findings, intracellular triglyceride levels were reduced by treatment with either WY-14643 or BI-9564 alone in both cell types and were further decreased by their combined treatment (Fig. 6D, E). These results suggest that BI-9564 suppresses lipid droplet formation by enhancing lipid metabolism through the induction of PPARα target genes.

To investigate the effect of Brd9 inhibition on fatty acid β-oxidation and hepatic lipid accumulation in vivo, WY-14643 and BI-9564 were intraperitoneally administered to C57BL/6J mice once a day for 4 days (Fig. 6F). Hepatic mRNA levels of Pparα target genes, including Cpt1a, were significantly increased by administration of either BI-9564 or WY-14643; however, no further enhancement was observed upon co-administration (Fig. 6G). Plasma triglyceride concentration was significantly decreased by BI-9564 or WY-14643, with the lowest levels observed in the co-administered group (Fig. 6H). Plasma NEFA levels tended to be lower with WY-14643 alone and were significantly decreased by the co-administration with BI-9564 (Fig. 6I). These results suggest that BI-9564 increases hepatic expressions of Pparα target genes and contributes to lowering plasma triglyceride and NEFA levels in vivo.

## Discussion

SWI/SNF complexes are large chromatin remodelers comprising 10–14 subunits, including the common subunits BRG1 and BAF60a. The physiological roles of SWI/SNF complexes have primarily been elucidated in the field of cancer biology (29). In addition, several studies have reported that the SWI/SNF complex regulates energy metabolism by regulating transcription factor function (17, 30). Our investigation aimed to elucidate the significance of SWI/SNF complexes in the transcriptional activation of PPARα, a key regulator of lipid metabolism.

The present study demonstrated that the inhibition of BRD9 using BI-9564 or the downregulation of BRD9 using siRNA increased CPT1A expression both with and without a PPARα agonist in hepatocytes (Figs. 1C–G and 3A–C). As with BRD9 knockdown, BRD7 knockdown in HepG2 cells significantly enhanced the PPARα-mediated *CPT1A* transcription (Fig. 3B, C). These findings prompted us to further consider the role of BRD7 in lipid metabolism. However, liver-specific knockout of Brd7 has been reported to increase lipid accumulation in high-fat diet-fed mice (31) and hepatic Brd7 expression was found to be lower in obese and diabetic model mice than in normal mice (32). BRD7 may thus have more potent effects on repressing the expression of lipid synthesis genes through mechanisms other than repression of PPARα-mediated transactivation of fatty acid oxidation genes. Alternatively, there may be species-specific differences in the target genes BRD7 between humans and mice. Taken together, further studies are required to elucidate the role of PBAF in hepatic lipid metabolism.

PPARα activation induces various genes involved in fatty acid oxidation, lipid transport, and anti-inflammatory responses. In this study, we found that BI-9564 enhanced the induction of several PPARα target genes, including CPT1A, PDK4, CPT2, SLC25A20, and FABP1, supporting BRD9’s role in promoting PPARα target gene expression (Fig. 2). While this trend was generally observed, some gene-specific variations emerged. For instance, PLIN2 induction was attenuated by BI-9564 in HepG2 cells, which may reflect differential regulatory mechanisms involving transcription factors beyond PPARα. For instance, PGC1α, a coactivator of CPT1A and PDK4 transcription, has been reported to interact with certain SWI/SNF complex subunits (12, 25). BRD9 may mediate the recruitment of PGC1α to the PPARα response element, thereby influencing chromatin remodeling and transcription. PLIN2 is known to be regulated by liver X receptor (LXR), which activates transcription of lipid biosynthesis genes (33). While it is uncertain whether LXR interacts with ncBAF, the high degree of amino acid sequence homology across nuclear receptors suggests that LXR may also interact with the SWI/SNF complex. The observation that some PPARα target genes were weakly induced by WY-14643 or BI-9564 alone but markedly upregulated upon WY-14643 and BI-9564 co-treatment indicates that BRD9 inhibition may potentiate PPARα activity or relieve transcriptional constraints. Thus, dissecting the chromatin-level mechanisms by which BRD9 modulates PPARα target gene expression will be crucial for identifying novel therapeutic strategies for lipid metabolism disorders, including metabolic dysfunction-associated steatotic liver disease (MASLD) and metabolic dysfunction-associated steatohepatitis (MASH), previously referred to as nonalcoholic fatty liver disease (NAFLD) and nonalcoholic steatohepatitis (NASH), respectively.

BRD7 and BRD9, components of SWI/SNF complexes, have been reported to interact with nuclear receptors, such as VDR (17), androgen receptor (34), and PXR (20), through the recognition of their acetylated lysine residues. In this study, immunoprecipitation using anti-His antibody revealed that both BRD7-His and BRD9-His interacted with FLAG-PPARα (Fig. 4A), whereas a pull-down assay revealed that the interaction of PPARα with BRD7 was weaker than that with BRD9 (Fig. 4B). These findings imply that PPARα interacts with ncBAF via BRD9 and may interact with PBAF via subunits other than BRD7. Suh *et al.* reported that human PPARα undergoes acetylation in HepG2 cells (35). Consistent with this, Western blot analysis using the anti-acetylated lysine antibody revealed that the FLAG-PPARα protein has an acetylated lysine(s) (Fig. 4C), suggesting that the interaction between PPARα and BRD9 is also mediated by acetylated lysine residue(s) of PPARα.

A plausible mechanism underlying the BI-9564-induced increase in CPT1A expression is the enhanced recruitment of PPARα to the intronic PPRE of the *CPT1A* gene. Our ChIP-qPCR analysis demonstrated that BI-9564 markedly increased PPARα binding at this site (Fig. 5B), suggesting that ncBAF inhibits PPARα binding to the *CPT1A* intronic PPRE. Supporting this interpretation, co-immunoprecipitation analysis revealed that the interaction between PPARα and BRD9 was attenuated by BI-9564 as well as WY-14643 (Fig. 5C). The conformation of PPARα protein is known to be changed by ligand binding (36). Such a conformational change in PPARα by binding of WY-14643 may interfere with the access of BRD9 to the acetylated lysine in PPARα. Because BI-9564 targets the bromodomain of BRD9 (37), the suppressed interaction between PPARα and BRD9 by BI-9564 may suggest that BRD9 directly interacts with PPARα via its bromodomain. Consistent with the attenuation of the interaction between PPARα and BRD9, we revealed that the chromatin accessibility around *CPT1A* intronic PPRE is significantly enhanced by WY-14643 and/or BI-9564 (Fig. 5D). These results suggest that BRD9 inhibition and PPARα activation cause relaxation of chromatin structure via dissociation of PPARα from ncBAF and enhance PPARα binding to *CPT1A* intronic PPRE.

Hepatic intracellular lipid accumulation causes organelle dysfunction, including endoplasmic reticulum stress, mitochondrial dysfunction, and lysosomal dysfunction, leading to cell death and progression to NASH (38). The formation of lipid droplets in hepatocytes is promoted by the uptake of FFAs and de novo lipogenesis, whereas it is inhibited by fatty acid oxidation and by export into blood as very low-density lipoprotein particles (39). As knockdown of Cpt1a dramatically increases lipid accumulation in the liver of mice fed a high-fat diet (40), Cpt1a may be a major regulator of lipid accumulation in the liver. Our results showed that treatment with WY-14643 and/or BI-9564 increased CPT1A expression (Fig. 1C–G) and significantly attenuated FFA-induced lipid accumulation (Fig. 6B, C). Moreover, consistent with the enhanced induction of PPARα target genes, co-treatment with BI-9564 and WY-14643 led to the most pronounced reduction in intracellular triglyceride levels (Fig. 6D, E), highlighting a synergistic effect on lipid metabolism. Recently, Li *et al.* (41) showed that the BRD7/9 co-inhibitor BI-7273 decreased hepatic lipid accumulation by inhibiting the activation of sterol regulatory element binding protein 1 via the Akt/mammalian target of rapamycin pathway. Because the activation of PPARα is known to attenuate Akt phosphorylation (42), the attenuation of lipid accumulation by BI-9564 may be mediated by inhibition of lipid synthesis, in addition to the fatty acid oxidation by CPT1A induction.

Following in vitro experiments, an in vivo study was performed to examine the effects of BI-9564 on lipid metabolism. Interestingly, co-administration of BI-9564 and WY-14643 did not result in a further increase in the expression of multiple Pparα target genes, including Cpt1a, Cpt2, Slc25a20, and Fabp1, compared to WY-14643 alone (Fig. 6G, H). It is possible that the binding of PPARα to *Cpt1a* PPRE was saturated because of exposure of the mouse liver to a higher concentration of WY-14643 (Fig. 6) than that of PMH (Fig. 1F). Nevertheless, the crucial finding is that BI-9564 alone was sufficient to induce a coordinated upregulation of these PPARα target genes. PPARα activation affects the expression of various genes involved in lipid metabolism, and the lipid-lowering effects by WY-14643 and/or BI-9564 (Fig. 6H) are presumed to reflect the combined actions of multiple PPARα downstream genes. The upregulation of Cpt1a and related genes, which play a central role in fatty acid oxidation (43), appears to contribute to the lipid-lowering effects of BI-9564. As WY-14643 is a synthetic PPARα agonist used in experimental settings (44), it remains unclear whether BI-9564 can enhance the transactivity of human PPARα activated by more physiologically relevant ligands such as endogenous fatty acids or clinically used fibrates. Plasma NEFA levels have been reported to be elevated in patients with obesity or NAFLD (45, 46). As our study demonstrated that co-administration of WY-14643 and BI-9564 decreases plasma NEFA levels (Fig. 6I), co-administration of a PPARα agonist and BI-9564 has the potential to suppress the progression of MASLD and MASH. The triglyceride- and NEFA-lowering effects of BI-9564 are suggested to involve PPARα activation. To provide more definitive evidence for this involvement, genetic studies using PPARα or BRD9 knockout model mice would be informative.

In conclusion, we found that inhibition of ncBAF enhanced PPARα agonist-induced fatty acid β-oxidation and lipid accumulation reduction. BRD9 inhibitors can thus have therapeutic potential for the treatment of dyslipidemia, MASLD, and MASH.

## Conclusions

In this study, we found that inhibiting BRD9, a subunit of the ncBAF, relaxes chromatin structure, thereby enhancing the binding of PPARα to the regulatory element of the target gene. This enhanced binding of PPARα subsequently activates the transcription of target genes, including *CPT1A*. Functionally, this upregulation leads to reduced lipid accumulation in hepatocytes and lower plasma triglyceride levels in mice. These findings suggest that BRD9 negatively regulates PPARα-mediated lipid metabolism, and its inhibition could be a novel therapeutic strategy for treating dyslipidemia, MASLD, and MASH. Further research is required to understand the precise mechanisms and therapeutic potential of BRD9 inhibitors.

## Data availability

The data that support the findings of this study are available from the corresponding author upon reasonable request.

## Supplemental data

This article contains supplemental data.

## Conflict of interest

The authors declare that they have no conflicts of interest with the contents of this article.
