# Supplementary material for: Chromatin remodeler BRD9 represses transcription of PPARα target genes, including CPT1A to suppress lipid metabolism
Source: J Lipid Res. 2025 Aug 6;66(9):100874. doi: 10.1016/j.jlr.2025.100874 (PMC12444171; doi:10.1016/j.jlr.2025.100874)
Supplement: Supplementary Figure 1 [file mmc1.docx]

**Supplemental information**

Supplemental Fig. 1. Identification of PPARα-specific bands.

To validate the PPARα band, HepG2 cells were transfected with siRNA targeting PPARα (s10881) for 72 hours. Western blotting of the nuclear fractions was performed using two different anti-PPARα antibodies (from GeneTex and Abcam). The original blot shown in Fig. 1D is included for comparison.
